# Mammographic density among indigenous women in forested areas in the state of Amapá, Brazil: a cross-sectional study: Densidade mamográfica em mulheres indígenas de áreas da floresta do estado do Amapá, Brasil: estudo transversal

**DOI:** 10.1590/1516-3180.2016.0146150317

**Published:** 2017-07-31

**Authors:** José Mauro Secco, Simone Elias, Cristina Valletta de Carvalho, Ismael Dale Cotrim Guerreiro da Silva, Kátia Jung de Campos, Gil Facina, Afonso Celso Pinto Nazário

**Affiliations:** I MD, PhD. Researcher, Universidade Federal de São Paulo (Unifesp), São Paulo (SP), and Adjunct Professor, Universidade Federal do Amapá (Unifap), Amapá (AP), Brazil.; II MD, PhD. Researcher, Universidade Federal de São Paulo (Unifesp), São Paulo (SP), Brazil.; III BSc, PhD. Researcher, Universidade Federal de São Paulo (Unifesp), and Adjunct Professor, Department of Biological Sciences, Centro Universitário Fundação Santo André, and Department of Genetics, Fundação ABC, São Paulo (SP), Brazil.; IV MD, PhD. Researcher, Universidade Federal de São Paulo (Unifesp); Adjunct Professor and Coordinator of Molecular Gynecology Laboratory, Department of Gynecology; and Coordinator of Research and Technological Innovation within Biology, Universidade Federal de São Paulo (Unifesp), São Paulo (SP), Brazil.; V MD, PhD. Researcher, Universidade Federal de São Paulo (Unifesp), São Paulo (SP), and Attending Physician and Residency Coordinator, Department of Gynecology, Universidade Federal do Amapá, Amapá (AP), Brazil.; VI MD, PhD. Full Professor, Department of Gynecology and Head of Department of Mastology, Universidade Federal de São Paulo (Unifesp), São Paulo (SP), Brazil.; VII MD, PhD. Researcher and Full Professor, Universidade Federal de São Paulo (Unifesp), São Paulo (SP), Brazil.

**Keywords:** Polymorphism, genetic, Receptors, estrogen, Receptors, progesterone, Health Services, indigenous, Population groups, Breast neoplasms

## Abstract

**CONTEXT AND OBJECTIVE:**

There is no register of breast cancer cases among indigenous populations in Brazil. The objective here was to evaluate the association of clinical and demographic characteristics with mammographic density among indigenous women.

**DESIGN AND SETTING:**

Cross-sectional analytical study conducted in indigenous territories in the state of Amapá, Brazil.

**METHODS:**

Women were recruited from three indigenous territories and underwent bilateral mammography and blood collection for hormonal analysis. They were interviewed with the aid of an interpreter. Mammographic density was calculated using computer assistance, and was expressed as dense or non-dense.

**RESULTS:**

A total of 137 indigenous women were included in this study, with an average age of 50.4 years, and an average age at the menarche of 12.8 years. Half (50.3%) of the 137 participants had not reached the menopause at the time of this study. The women had had an average of 8.7 children, and only two had never breastfed. The average body mass index of the population as a whole was 25.1 kg/m^2^. The mammographic evaluation showed that 82% of women had non-dense breasts. The clinical characteristics associated with mammographic density were age (P = 0.0001), follicle-stimulating hormone (FSH) (P < 0.001) and estrogen levels (P < 0.01).

**CONCLUSIONS:**

The majority of the indigenous women had non-dense breasts. Age, menopausal status and FSH and estrogen levels were associated with mammographic density.

## RESUMO

**CONTEXTO E OBJETIVO:**

Não há registro de casos de câncer de mama em populações indígenas no Brasil. O objetivo foi avaliar a associação de características clínicas e demográficas com a densidade mamográfica em mulheres indígenas.

**TIPO DE ESTUDO E LOCAL:**

Estudo transversal, analítico, realizado em territórios indígenas no estado do Amapá, Brasil.

**MÉTODOS:**

Mulheres foram recrutadas de três territórios indígenas e submetidas a mamografia bilateral e a coleta de sangue para análise hormonal. As participantes foram entrevistadas com a ajuda de um intérprete. A densidade mamográfica foi calculada com assistência de computador, e expressa como densa ou não densa.

**RESULTADOS:**

137 mulheres foram incluídas no estudo, com média de 50,4 anos e média de idade à menarca de 12,8 anos. Metade (50,3%) das 137 participantes não havia entrado na menopausa no momento do estudo. As mulheres tinham em média 8,7 filhos, e duas nunca haviam amamentado. O índice de massa corpórea médio da população como um todo foi de 25,1 kg/m^2^. A análise mamográfica mostrou que 82% das mulheres tinham mamas não densas. As características clínicas associadas com a densidade mamográfica foram idade (P = 0.0001), hormônio folículo-estimulante (FSH, P < 0,001) e níveis de estrogênio (P < 0,01).

**CONCLUSÃO:**

A maioria das indígenas tinha mamas não densas. Idade, *status* menopausal e níveis de estrógeno e FSH foram associados com a densidade mamográfica.

## INTRODUCTION

It is known that breast cancer is less prevalent among African-American women than among white women. However, the disease onset is earlier among African-Americans, and these women have more aggressive tumors.^1^^,^^2^ Women of indigenous origin clearly present lower incidences of breast cancer than women with no indigenous descent.^3^^,^^4^^,^^5^^,^^6^

The incidence of breast cancer in the indigenous population in Brazil is very low, and absolutely no cases have been found in some ethnic groups, such as the Xavantes group, living in the state of Mato Grosso.^7^ This phenomenon can be explained by lifestyle habits: some peculiarities of indigenous populations are indeed protective factors, e.g. first pregnancy early in life, multiparity, prolonged breastfeeding and absence of hormone therapy. The hypotheses for explaining the lower incidence of breast cancer among indigenous women may include lower life expectancy and underreporting.^7^ However, indigenous populations also present some risk factors for breast cancer: sedentary lifestyle, overweight and obesity are observed in more than half of these women.^3^

Breast density, as evaluated by mammography (mammographic density), is an independent risk factor for breast cancer, which is independent of age, menopausal status or exogenous steroid use.^8^^,^^9^^,^^10^^,^^11^^,^^12^ To our knowledge, there is no other study in the literature addressing the relationship between demographic and clinical factors and mammographic density among indigenous women in Brazil.

## OBJECTIVE

This study aimed to evaluate clinical and demographic characteristics and their association with mammographic density among indigenous women.

## METHODS

### Study design, participants and ethics

In this cross-sectional analytical study, indigenous women in Oiapoque, in the Brazilian state of Amapá, were evaluated. These women were recruited in three indigenous territories, Uaça, Galiby and Juminã, which are home to four indigenous ethnicities: Karipuna, Galibi Marworno, Palikúr and Galibi Kalina, distributed in 38 villages. The total population of these indigenous territories in 2010 was 7,021, according to figures from the local technical coordination office of the National Indian Foundation, in Oiapoque.

To evaluate these women, it was necessary to obtain authorizations from the indigenous leaders of Oiapoque, from the National Indian Foundation (FUNAI) and from the National Health Foundation (FUNASA). Permission from the indigenous leaders was obtained for this study after we participated in the annual meeting of the Association of Indigenous Peoples of Oiapoque (APIO), at which the research project was presented to the community. An informed consent form was signed by all the women to be examined, after the study objectives had been explained to them by a local interpreter, in the local Indian Community Center during the interview. Ethical approvals were obtained from the National Committee for Research Ethics (CONEP) and the local Ethics Committee of the university hospital.

The inclusion criteria were that the women needed to be 40 years of age or older, living in indigenous villages (and not in cities) and not using hormonal medications (including plant hormones) to treat menopausal symptoms during the 12 months preceding the interview. Women suffering from endocrine, liver or kidney disorders, as ascertained from the local medical records kept by FUNAI and FUNASA, were also excluded. Mammograms that were classified as Breast Imaging Reporting and Data System (BI-RADs) category 3 or above also constituted an exclusion criterion *a priori*.

From January to December 2009, it was possible to obtain authorizations and arrange transportation to examine 150 indigenous women. All of them, except for nine with age disparities and two with BI-RADs category 3, underwent blood collection for follicle-stimulating hormone (FSH) and estradiol tests and underwent mammography.

The subjects were transported by bus to the nearest big city to undergo mammography exams. Because they live in villages far from each other, the women were transported by boat or car from their villages to the Indian House, in the municipality of Oiapoque, and from this place they were transported by bus, for about 450 km, to another Indian House, in the city of Macapá, where they were interviewed by the principal investigator and a nurse technician. After the women had signed the informed consent form, blood samples were collected from them to assay for FSH and estradiol (E2). About 5 ml of peripheral blood were obtained from each subject, using a vacuum extraction tube containing the anticoagulant EDTA (ethylenediaminetetraacetic acid). For mammography, the women were then transported to Hospital São Camilo in Macapá.

All the women included in this study underwent analogue bilateral mammography on the same device (Lorad Affinity, with Kodak M35 X-OMAT processor and Kodak Min-R 2000 film). Two projections were obtained for each breast: mediolateral oblique (MLO) and craniocaudal (CC). The same radiologist who performed the examinations made the first classification of cases in accordance with the BI-RADS criteria, for confirmation of enrollment in the study. Women with mammographic scans in BI-RADS categories 1 and 2 of the American College of Radiology (ACR), i.e. indicating absence of changes suggestive of breast cancer, were included in this study. We then calculated mammographic density using the method described below.

### Mammographic density evaluation

Firstly, two independent examiners evaluated the scans to determine the mammographic density. They worked subjectively, based on the mammographic patterns described in the ACR’s BI-RADS 2003 manual.^1^ The ratings D1, D2, D3 and D4 were grouped two-by-two to enable statistical analysis: D1 + D2 were considered to represent non-dense breasts and D3 + D4, dense breasts.

The mediolateral oblique incidence scans were then digitized (scanner CX312.T, Radiographic Digital Imaging, Compton, CA, USA). Following this, a third evaluator assessed mammographic density by using computer software for image analysis. Mammographic density was calculated using the gray-scale histogram tool of the Adobe Photoshop CS3 version 10.0 software as follows. The kappa coefficient was used to calculate the concordance between the three observers, two-by-two. Because the concordance was high (r > 0.78; P < 0.001), the third observer (called “computer-assisted evaluation” henceforth) was used as reference from then on.

Mammographic density was objectively determined using imaging analysis computer software. By convention, the mid-lateral left oblique incidence was scanned and captured using a CX312.T scanner (Radiographic Digital Imaging, Compton, CA, USA).

**Figure 1.**
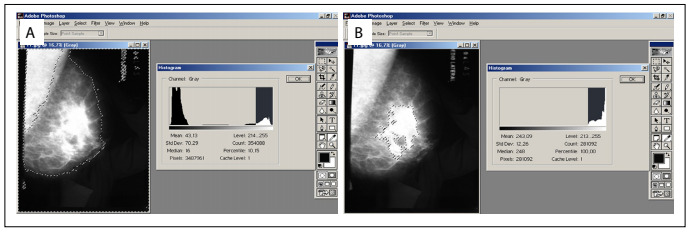
(A) Use of the “Lasso Tool” in Adobe Photoshop software, on a mammogram scan, for selection of the area to be calculated; and (B) “Magic Tool” for selection of dense tissue.

Initially, using the “Lasso Tool” (Figure 1), the image of the entire breast was selected, taking care to exclude the pectoral muscle. Through this procedure, the software generated a numerical value corresponding to the number of pixels in the overall breast area. Then, using the “Magic Tool”, the densest area of the breast, corresponding to fibroglandular tissue, was selected to obtain the number of pixels of this area. Finally, the Equation 1 was applied, to determine the percentage of fibroglandular tissue:

$$\;Mammographic\;density\;(MD)\;=\frac{dense\;area\;(DA)\times 100}{total\;area}$$ (1)

The values obtained for the dense area were compared with the subjective values registered by the other two evaluators, and the concordance between them was calculated using the kappa coefficient.

### Statistical analysis

The concordance between the three observers was calculated by means of the kappa coefficient, two-by-two. Because the concordance was high (kappa > 0.75), the evaluations of the third observer (henceforth referred to as “computer-assisted evaluation”) were used as a reference. To decrease the number of classes within the computer-assisted evaluation variable, mammogram patterns were added as follows: D1 + D2 with the group of non-dense breasts; and D3 + D4 with the group of dense breasts.

The chi-square test was used for comparing qualitative variables (frequency and proportions). To compare quantitative data (mammographic density), the Mann-Whitney U test was used where necessary. When the expected frequency was less than 5, we used Fisher’s exact test. To calculate odds ratios and confidence intervals, binary logistic regression was performed. Statistical significance was set at 5% or P < 0.05. The statistical software SPSS (Statistical Package for the Social Sciences), version 14.0, was used.

## RESULTS

**Figure 2.**
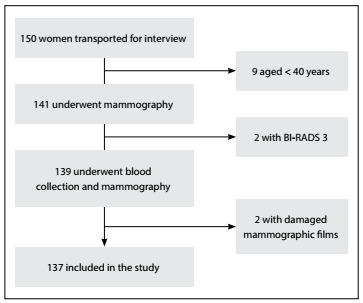
Flowchart of patients’ recruitment and inclusion in the study.

At the beginning of the study period, 150 women were recruited in the indigenous villages and were transported to undergo mammography. However, it was found that 9 of them were actually younger than 40 years, which prevented their inclusion in the study. Mammography was performed on the remaining 141 women, but 2 of them were classified as BI-RADS 3, which probably indicated benign lesions, for which follow-ups six months afterwards were suggested. Nevertheless, because this finding also constituted an exclusion criteria of the study, these 2 women were excluded. Thus, a total of 139 indigenous women whose mammograms showed no signs of breast lesions were initially included in the study. However, in two cases, the mammogram film was damaged by moisture before it could be digitized for the analysis on mammographic density (Figure 2). The average age of the remaining 137 women initially included was 50.4 years, with the menarche at an average of 12.8 years (the earliest was at 10 years of age). About half (50.3%) of the women were premenopausal.

Only 10 of the women (0.7%) had a history of using hormonal contraceptives. Only one of them did not have any children. The total number of offspring of the 138 women who had children was 1,209, an average of 8.7 per mother; but more than half of them (57.2%) had 9 or more children, and one of them had 19.

The average age at which they had their first child was 15.4 years, and this ranged from 12 years of age (three women) to 22 years of age. All of the women except for five had had children before reaching 18 years of age. Half of them (50%) had had children before the age of 15.

Only two women (1.4%) had never breastfed: one without children and the other with six children. Ninety-eight women (70.5%) had breastfed for a minimum of 10 years. In relation to body mass index (BMI), 66 (47.4%) had a BMI indicating that they were overweight (≥ 25 kg/m^2^). The average BMI of the population as a whole was 25.1 kg/m^2^. None of the women interviewed had any history of alcoholism (defined as the equivalent of two doses of distilled spirits per day); 44 of them reported occasional alcohol use (31.6%), which was only at annual community festivals.

Most (68%) of the indigenous women were from two villages: Kumarumã (the Galibi Marworno people) and Espírito Santo (the Karipuna people). However, neither the subjects’ tribe nor their village showed any significant association with any of the other variables of this study. Alcoholism (characterized as the equivalent of two servings of distilled liquor per day) was nonexistent in the sample (only occasional consumption of alcohol could be verified), which prevented calculation of any association with mammographic density. Nor did the distribution of origin (town or village) show any association with the main variables of the study. The Shapiro-Wilk test revealed that the sample data did not have normal distribution regarding the sociodemographic variables, so the Mann-Whitney test was used for the mammographic density association tests (as described below).

### Mammographic density

Among the 137 women for whom evaluation of mammographic density was possible, the distribution of the evaluations was classified as D1, D2, D3 and D4 (D = mammographic density). Table 1 shows the number of evaluations made by each observer and the final average. As shown in Table 2, the three observations (observers 1, 2 and the computer-assisted) were highly correlated (coefficient r > 0.8, with P < 0.001).

**Table 1.**
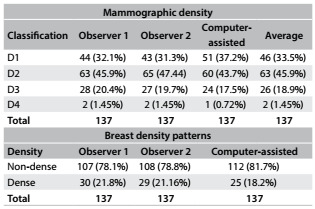
Distribution of mammographic density according to the observers

D1 to D4 refer to breast density classification; the least dense breast is D1 and most dense is D4.

**Table 2.**
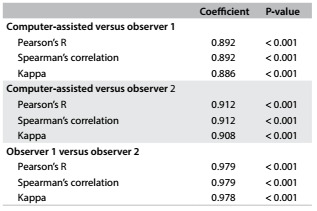
Agreement between observers, according to kappa coefficient, considering dense and non-dense mammographic densities together

The D1, D2, D3 and D4 classifications were gathered into two groups to enable statistical analysis: thus, D1 + D2 were considered to be non-dense breasts and D3 + D4 to be dense breasts. Hence, as shown in Table 1, the majority of the cases were of non-dense breasts. The inter-observer concordance was also high when the classifications were grouped (D1 + D2) and (D3 + D4) (Table 3).

**Table 3.**
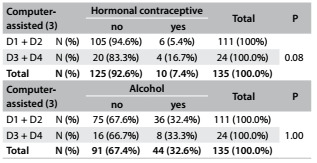
Descriptive statistics on the frequency of hormonal contraceptive and alcohol use, and comparison with mammographic density using Fisher’s exact test

### Mammographic density and association with other variables

Considering the high agreement (kappa) between the observers, we started to investigate associations with other variables, using the computer-assisted evaluation as a standard. Mammographic density was not significantly associated with any history of hormonal contraceptive use (P = 0.08) or with alcohol consumption (P = 1.00). Several other variables were also not associated with mammographic density (Table 4).

**Table 4.**
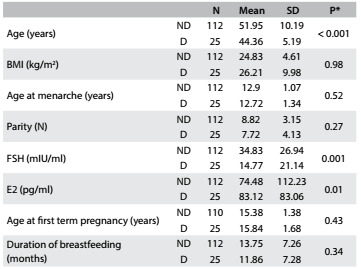
Clinical and hormonal characteristics of the indigenous women with non-dense (ND) and dense (D) breasts

SD = standard deviation; BMI = body mass index; FSH = follicle-stimulating hormone; E2 = estradiol. *Mann-Whitney (z) test for non-parametrical variables.

Two variables were significantly associated with mammographic density: serum levels of follicle-stimulating hormone (FSH) and estradiol (E2). Higher levels of FSH were associated with non-dense breasts (P = 0.001) and higher levels of E2, to dense breasts (P = 0.01). The participants’ average age was significantly associated with mammographic density (Table 4).

There were 69 postmenopausal women and 68 were premenopausal. Most of the postmenopausal women had non-dense breasts (67; or 97%). Dense breasts were seen in two women after the menopause (3%) and 23 before (34%). The mammographic density was associated with the menopausal status (Table 5; P < 0.0001).

**Table 5.**
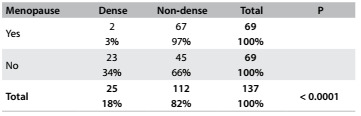
Distribution of dense and non-dense breasts according to menopausal status

## DISCUSSION

There is a high incidence of certain types of malignant tumors, such as colon, uterine and gastric cancers, in the Amazon region. However, absence of breast cancer among the indigenous women of several Brazilian states, such as Mato Grosso, Mato Grosso do Sul and Paraná, has been reported in the literature.^3^^,^^6^^,^^7^ The National Health Foundation (FUNASA) keeps up-to-date records of the diseases that occur in the indigenous population, which undermines the hypothesis that underreporting is responsible for non-registration of cases of breast cancer among indigenous women in Brazil.^13^ In the international literature, lower incidence of breast cancer has been reported in indigenous than in non-indigenous populations in the United States^14^^,^^15^ and extremely low incidence was reported among indigenous women in Ecuador.^5^ Breast cancer is more prevalent among women living in large cities.^2^ Because dense breasts are an independent risk factor for breast cancer (and this risk persists for 10 years or more^16^), lower mammographic density might be a protective factor against breast cancer in indigenous populations.^17^^,^^18^ That hypothesis inspired the present investigation, which was undertaken among women living in indigenous villages in the Amazon forest, far from urban areas. Because of this, these women were probably not subject to the lifestyle and dietary modifications seen in urban regions.

Several studies have shown lower mammographic density in women of indigenous ethnicity. This may have been due not only to their reproductive pattern of bearing many children, with prolonged periods of breastfeeding, but also perhaps to their indigenous ethnicity itself. The indigenous women of New Mexico, United States, have early liposubstitution of the breasts, compared with Hispanic and non-Hispanic white women.^19^^,^^20^ Roubidoux et al. also observed lower mammographic density among indigenous women in the southwestern United States.^15^ These authors also made comparisons between different ethnic groups in Alaska and observed that indigenous and Aleut women had less dense breasts than Eskimos.^21^ In all of these studies, lower breast density was associated with lower incidence of breast cancer. In the present study, we confirmed this finding: the majority of the cases were indeed of non-dense breasts: the mammographic density in our study was well below what has been reported in the literature for non-indigenous populations.^22^^,^^23^^,^^24^

The importance of accurate determination of mammographic density was emphasized by Boyd et al.,^25^ who observed a 2% increase in the relative risk of breast cancer for each 1% increase in the percentage of mammographic density. We sought to conduct an objective evaluation in the present study by scanning and capturing one of the mammographic views and determining the percentage area of fibroglandular tissue, and consequently, the mammographic density. The lower subjectivity was due to the calculation method, but the definition of the area to be calculated remained subjective, since it was demarcated using the computer mouse. In our study, when we grouped D1 + D2 as non-dense breasts and D3 + D4 as dense breasts, we had a high degree of inter-observer concordance, of 0.97 (< 0.0001). This level of concordance also resulted from the care with which the images were obtained, using the same technical process in all cases.

In this study, the association between age and mammographic density was statistically significant (P ≤ 0.001). It has been demonstrated in the literature that breast tissue becomes replaced by fat with advancing age. Nonetheless, the mammographic density in our study was much lower than that of non-indigenous populations.^15^^,^^25^^,^^26^^,^^27^ In our series, the frequency of non-dense breasts was 82%; and among the 69 postmenopausal women, only two (3%) had dense breasts, with an average age of 50.4 years. In non-indigenous populations in Brazil, three studies have shown that approximately 30% of women aged 50 years and over who were not using hormonal contraceptives had dense breasts.^22^^,^^23^^,^^24^ In another Brazilian study, in which the participants had a mean age of 54 years, the frequency of observation of dense breasts was 45%.^18^ In Sweden, Bergkvist et al.^28^ observed that 65% of the women aged 35 years had high mammographic density and 15-20% of those aged 55 years.

Mammographic density is influenced by several factors, such as reproductive history, BMI, hormonal patterns and genetic factors.^29^ In the present study, only age, menopausal status and FSH and estradiol levels were associated with mammographic density. Indigenous women with dense breasts had lower FSH levels and higher estradiol levels than those with non-dense breasts. The estradiol levels in postmenopausal women were equivalent to those observed in other studies,^29^^,^^30^ which concluded that in postmenopausal women, mammographic density was inversely related to estradiol levels.

First childbirth before the age of 24 years, having more than two children and breastfeeding for more than two years are considered by many authors to be protective factors against breast cancer.^3^^,^^21^^,^^27^ Reproductive behavior is quite different in indigenous populations, and this probably contributes towards creating a protective effect against breast cancer. This, together with other factors, might explain the low incidence of this disease among these women. Both in our study and in others, the number of children per indigenous woman was high, and the first delivery happened at an early age, thus resulting in many years of breastfeeding.^3^^,^^21^^,^^27^^,^^30^ Parity is inversely associated with mammographic density,^31^ and thus represents a protective factor against breast cancer.^32^ It seems that the state of involution depends, in part, on parity: after successive pregnancies, stem cells and/or progenitor cells would accumulate in the mammary glands, and this has been observed in multiparous female mice. This is a valid hypothesis that would also explain the relationship between density and parity.^33^

Lactation has consistently been inversely correlated with the risk of breast cancer: the risk decreases by 4.3% for every 12 months of breastfeeding.^34^ Currently, the relationship between breastfeeding and mammographic density is a matter of controversy, given that both positive and inverse associations have been found.^32^^,^^35^^,^^36^^,^^37^ The women in our study nursed for a long period (mean of 12.9 years), and their breasts were predominantly non-dense, but despite this, breastfeeding was not significantly associated with mammographic density.

One limitation to consider in our study is that, due to the difficulties in conducting the study in this scenario of remote indigenous populations in the Amazon region, it was not possible to ascertain whether these participants might have any other clinical conditions, such as endocrine, hepatic or renal diseases. However, we ruled out the presence of these diseases based on data in the medical records provided by the National Health Foundation (FUNASA), which was the institution responsible for healthcare among indigenous people until 2010. The main researcher (JMS) himself analyzed data collected by FUNASA and observed that there was an electronic information system regarding indigenous people’s health: Sistema de Informação da Saúde Indígena (SIASI). This was constantly updated with data from the special indigenous health districts (Distritos Especiais de Saúde Indígena, DISEIS). Full healthcare information is registered in this database, including international classification of diseases (ICD) codes, and the information was considered to be quite reliable. Hence, it is unlikely that these women were suffering from diseases that could interfere with the results from the present study.

## CONCLUSIONS

In this population of indigenous women in the municipality of Oiapoque, in the state of Amapá, Brazil, there were no cases of breast cancer and mammographic density was predominantly low. Age, menopausal status and FSH and estrogen levels were associated with mammographic density.
